# Anisotropic and nonlinear magnetodielectric effects in orthoferrite ErFeO_3_ single crystals

**DOI:** 10.1038/s41598-020-68800-x

**Published:** 2020-07-16

**Authors:** Dong Gun Oh, Jong Hyuk Kim, Hyun Jun Shin, Young Jai Choi, Nara Lee

**Affiliations:** Department of Physics, Yonsei University, Seoul, 03722 Korea

**Keywords:** Physics, Condensed-matter physics, Ferroelectrics and multiferroics, Magnetic properties and materials

## Abstract

In rare-earth orthoferrites, strongly correlated order parameters have been thoroughly investigated, which aims to find multiple functionalities such as multiferroic or magnetoelectric properties. We have discovered highly anisotropic and nonlinear magnetodielectric effects from detailed measurements of magnetoelectric properties in single-crystalline orthoferrite, ErFeO_3_. Isothermal dielectric constant varies in shapes and signs depending on the relative orientations between the external electric and magnetic fields, which may be ascribed to the spin-phonon couplings. In addition, a dielectric constant with both electric and magnetic fields along the *c* axis exhibits two symmetric sharp anomalies, which are closely relevant to the spin-flop transition, below the ordering temperature of Er^3+^ spins, *T*_Er_ = 3.4 K. We speculate that the magnetostriction from the exchange couplings between Er^3+^ and Fe^3+^ magnetic moments would be responsible for this relationship between electric and magnetic properties. Our results present significant characteristics of the orthoferrite compounds and offer a crucial guide for exploring suitable materials for magnetoelectric functional applications.

## Introduction

Research on novel magnetic materials aims to understand the relationship between microscopic magnetic orders and macroscopic physical phenomena along with the development of desired functional properties. Magnetic oxides composed of metal cations and oxygen anions have been widely explored because of the abundance of constituents and stability of compounds. In particular, some of the materials exhibit multiferroicity^1,2^ and magnetoelectricity^3,4^, which are characterized by cross-couplings between electric and magnetic order parameters. Such intriguing aspects provide a beneficial foundation for technological applications such as magnetoelectric data storage and sensors^5–9^. Recently, rare-earth orthoferrites (RFeO_3_; R: rare-earth ions) have received tremendous attention for materials research due to ultrafast spin switching^10–12^, large magnetocaloric effect^13,14^, reversible magnetic exchange-bias^15,16^, and magnetism-driven ferroelectricity^17,18^. In DyFeO_3_, a giant magnetoelectric tensor component is observed, and electric polarization is found to be reversible by switching the direction of the magnetic field^17^. In GdFeO_3_, the multiferroicity emerges below *T*_Gd_ = 2.5 K due to the symmetric exchange striction between Gd^3+^ and Fe^3+^ magnetic moments^18^. In addition, magnetoelectric domains, in which ferroelectric and canted-antiferromagnetic domain walls are strongly fastened, lead to cross-controls of electric polarization and magnetization by applying magnetic and electric fields, respectively.

Although the potential multiferroicity was suggested in ErFeO_3_ (EFO)^19^, no direct evidence has yet been presented. In a polycrystalline EFO, the dielectric responses with strong frequency dependence were observed in a broad temperature range^20^. For example, thermally-activated dielectric relaxation, ascribed to the polaron relaxation arising from carrier hoppings between Fe^2+^ and Fe^3+^ ions, was found at approximately 200 K, and relaxor-like broad dielectric anomalies was also presented at approximately 550 K. The ferroelectricity arising below *T*_Gd_ = 2.5 K in GdFeO_3_ suggests that the additional ordering of rare-earth ions in orthoferrites would provide a substantial modification to the magnetic properties, which are imperative when determining new functional characteristics. In this study regarding magnetoelectric properties in single-crystalline EFO, we find an absence of ferroelectricity but reveal strongly anisotropic and nonlinear magneto-dielectric effects below *T*_Er_ = 3.4 K. The magnetodielectric (MD) behavior exhibits versatile magnetic-field dependences, which are possibly ascribed to the spin-phonon couplings.

## Results and discussion

**Figure 1:**
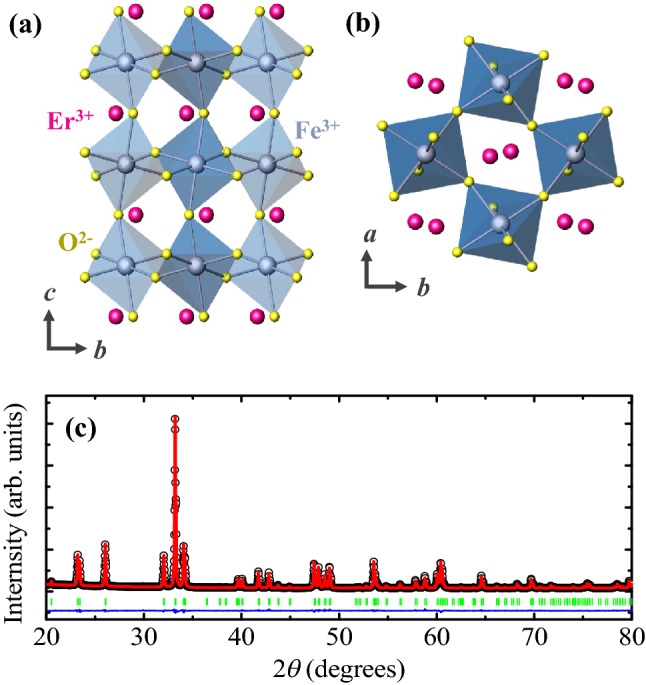
Crystallographic structure of ErFeO_3_. (**a**) and (**b**) Schematics of the crystallographic structure of perovskite ErFeO_3_ (EFO) from the *a* and *c* axes, respectively. The pink, grey, and yellow spheres represent Er^3+^, Fe^2+^, and O^2−^ ions, respectively. (**c**) Observed (open circles) and calculated (solid line) powder X-ray diffraction patterns for the ground EFO single crystals. The blue curve represents the intensity difference between the observed and calculated patterns. The green short lines denote the Bragg positions.

Figure 1a and b depict the crystallographic structures of EFO viewed from the *a*- and *c*-axes, respectively. The corner-shared octahedral units of Fe^3+^ ions are strongly distorted due to the small radius of the Er^3+^ ions. A detailed structure was obtained from the Rietveld refinement using the Fullprof Suite program for the X-ray diffraction pattern of the ground EFO, measured at room temperature. In Fig. 1c, the observed and calculated patterns are shown as open circles and solid lines, respectively. As suggested from the result, the EFO crystallizes in a *Pbnm* orthorhombic structure with lattice parameters, *a* = 5.2611 Å, *b* = 5.5835 Å and *c* = 7.5915 Å, with an agreement factor of *χ*^2^ = 3.29 (see Supplementary Information S1 for details).

**Figure 2:**
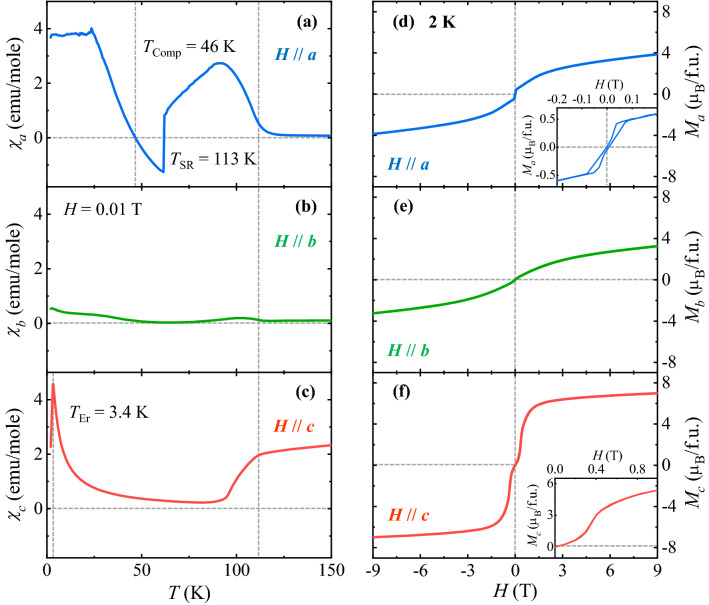
Temperature- and magnetic-field-dependent magnetic properties of ErFeO_3_. (**a**)–(**c**) Temperature dependence of magnetic susceptibility, *χ* = *M*/*H*, shown for the EFO crystal for *H* = 0.01 T upon warming after zero-magnetic-field cooling along the *a*, *b*, and *c* axes, respectively. The vertical dotted lines indicate the spin-reorientation temperature (*T*_SR_ = 113 K), compensation temperature (*T*_comp_ = 46 K), and ordering temperature of Er^3+^ moments (*T*_Er_ = 3.4 K). (**d**)–(**f**) Isothermal magnetization of the EFO crystal in *H*//*a* (**d**), *H*//*b* (**e**), and *H*//*c* (**f**), measured at *T* = 2 K up to 9 T. The inset of (**d**) shows the magnified view in the range of *H* =  ± 0.2 T of the hysteresis loop in *H*//*a*. The inset of (**f**) displays the enlarged view in the rage of *H* = 0–1 T in *H*//*c*.

The anisotropic magnetic properties of EFO were measured by the *T* dependence of magnetic susceptibility (*χ* = *M/H*) at *H* = 0.01 T for *a*, *b*, and *c* axes upon warming after the zero-field-cooling process, as shown in Fig. 2a–c. The canted antiferromagnetic ordering of Fe^3+^ magnetic moments is known to occur at *T*_N_ ≈ 640 K^21,22^, below which the net magnetic moment becomes aligned along the *c* axis, and starts to rotate into the *a* axis by 90° at *T*_SR_ = 113 K, evidenced with a significant increase of *χ*_*a*_ (Fig. 2a) and a reduction of *χ*_*c*_ (Fig. 2c) below *T*_SR_. In Fig. 2c, after completing the spin reorientation at approximately 93 K, *χ*_*c*_, upon further cooling increases smoothly. The sharp peak of *χ*_*c*_ at *T*_Er_ = 3.4 K indicates the long-range antiferromagnetic ordering of Er^3+^ magnetic moments aligned along the *c* axis. According to previous studies, the Er^3+^ spins are also canted along the *a* axis and their larger net magnetic moment tends to align in the opposite direction of the net moment of Fe^3+^ spins^19,23–25^. In Fig. 2a, the net moment of the Er^3+^ sublattice at a low *T* regime follows the direction of the applied magnetic field while the smaller net moment of the Fe^3+^ sublattice is aligned in the opposite direction. As *T* is increased, the dominant ferrimagnetic ordering between the Er^3+^ and Fe^3+^ sublattices is identified by the compensated magnetization at *T*_Comp_ = 46 K. Further decreasing the net moment for the Er^3+^ sublattice induces a sudden reversal of total magnetization at approximately 61 K. In Fig. 2b, the overall behavior of *χ*_*b*_ reveals only weak *T* variation, which indicates that the Er^3+^ and Fe^3+^ spins do not tend to align along the *b* axis. It appears that the several different studies of magnetic properties on the single crystalline EFO reveal the sample dependence of magnetic transition temperatures^15,25,26^. In oxide compounds, oxygen contents vary in a broad range depending on the growth conditions and/or post annealing procedures in different gas environments, which would influence on the electronic and magnetic properties^27–30^. To verify the oxygen content of our EFO, we used an EPMA (Electronic Probe Micro-Analyzer, JEOL JXA-8530F). Each sample was measured at several points on the surface to confirm oxidation of the sample surface, which shows the oxygen content of the EFO crystals as 2.81. The oxygen deficiency on the crystal surface possibly results from the growth nature of flux method and may incorporate small amount of Fe^2+^ ions.

Isothermal magnetizations for the three different orientations were measured by applying *H* up to ± 9 T at *T* = 2 K. In the inset of Fig. 2d, *M*_*a*_ exhibits tiny magnetic hysteresis, consistent with the canted antiferromagnetism with the net magnetic moment along the *a* axis. The susceptible response of a small net magnetic moment leads to a narrow hysteresis loop with negligible amount of residual net magnetization and tiny coercive field as ~ 0.007 T, similar to the behavior of a soft ferromagnet. Upon further increasing *H*, *M*_*a*_ increases linearly up to approximately 1.3 T, whereas the slope slowly declines afterward. *M*_*a*_ at a maximum of *H* (9 T) is approximately 3.85 μ_B_/f.u. (Fig. 2d). *M*_*b*_ exhibits a smooth increase up to 9 T with a magnetization value of 3.25 μ_B_/f.u., without magnetic hysteresis (Fig. 2e). In Fig. 2f, *M*_*c*_ rises sharply at ~ 0.34 T, identified by a sharp peak in the *H* derivative of *M*_c_, which indicates the spin flop transition of the Er^3+^ and Fe^3+^ magnetic moments. The inset shows the enlarged view of *M*_*c*_, which indicates the absence of a hysteretic behavior at the spin flop transition. *M*_*c*_ increases gradually above 1.5 T and reaches the largest magnetic moment of 6.98 μ_B_/f.u. at 9 T.

**Figure 3:**
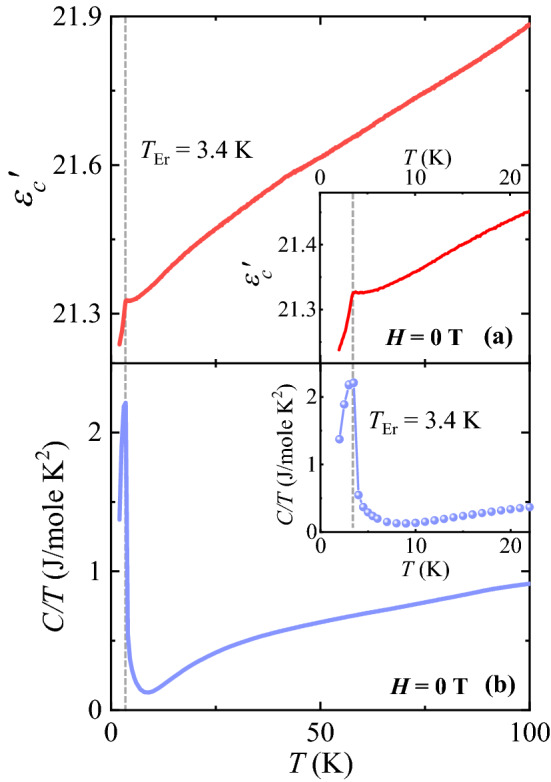
Temperature-dependent dielectric constant and specific heat. (**a**) Temperature dependence of dielectric constant, measured along the *c* axis (*ε*_*c*_′) at *H* = 0 T. Inset shows the low-temperature regime of *ε*_*c*_′. (**b**) Temperature dependence of the ratio of specific heat and temperature, *C*/*T*, measured at *H* = 0 T. Inset shows the low-temperature regime of *C*/*T*.

The *T*-dependence of *ε*′ is displayed in Fig. 3a, measured along the *c* axis (*ε*_c_′) at *f* = 500 kHz for *H* = 0 T. *ε*_*c*_′ decreases monotonously from a high *T* regime and exhibits an abrupt decrease below *T*_Er_ = 3.4 K that corresponds to the long-range ordering of Er^3+^ magnetic moments. This suggests that the intrinsic magnetic ordering of the Er^3+^ moments would influence strongly on *ε*_*c*_′ in the EFO. Additionally, the *T*-dependence of the heat capacity divided by the temperature (*C/T*) measured upon warming in zero *H* shows a sharp peak at *T*_Er_, (Fig. 3b). In contrast, no anomaly of *C/T* was observed at *T*_Comp_ in spite of the abrupt change of *χ*_*a*_ (Fig. 2a). It involves only a sign change of *χ*_*a*_ due to thermal fluctuation but is not relevant to an additional entropy change. In GdFeO_3_^18^ and DyFeO_3_^17^, multiferroicity and magnetic-field-induced ferroelectricity emerge along the *c* axis below the ordering temperatures of Gd^3+^ and Dy^3+^ ions, respectively, due to the symmetric exchange strictions. However, the clear anomaly of *ε*_*c*_′ at *T*_Er_ in the EFO does not involve a ferroelectric polarization in pyro- and magneto-electric current measurements, which indicates the absence of multiferroicity (see Supplementary Information S2 for details). A similar decreasing behavior in *ε'* was observed in Y_2_Cu_2_O_5_ below the *T* of antiferromagnetically ordered Cu^2+^ spins, *T*_N_ = 13.2 K^31^.

**Figure 4:**
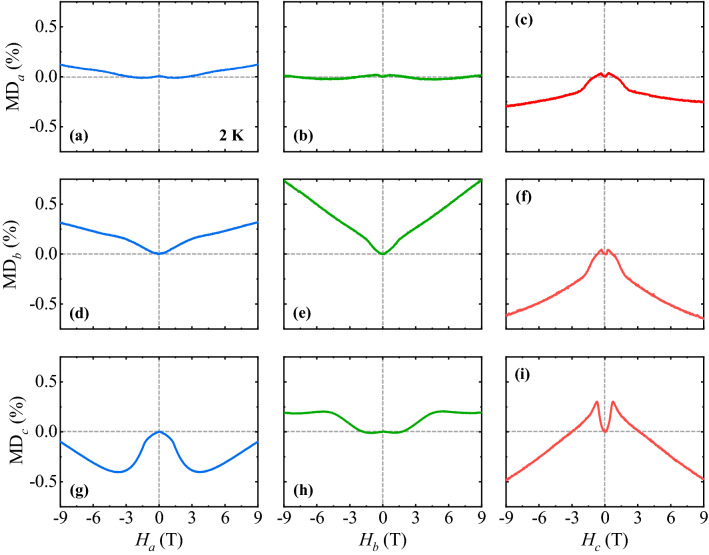
Magnetodielectric effect of ErFeO_3_. Magnetodielectric (MD) effect defined as MD (%) = $\frac{{\varepsilon^{{\prime }} \left( H \right) - \varepsilon^{{\prime }} \left( {0 {\text{T}}} \right)}}{{\varepsilon^{{\prime }} \left( {0 {\text{T}}} \right)}} \times 100$ for (**a**)–(**c**) *a* (MD_*a*_), (**d**)–(**f**) *b* (MD_*b*_), and (**g**)–(**i**) *c* (MD_*c*_) axes, respectively, at *H*_*a*_, *H*_*b*_ , and *H*_*c*_ up to ± 9 T and *T* = 2 K.

In Fig. 4, the MD effect, described by the variation of *ε'* by applying *H* and defined as MD (%) = $\frac{{\varepsilon^{{\prime }} \left( H \right) - \varepsilon^{{\prime }} \left( {0 {\text{T}}} \right)}}{{\varepsilon^{{\prime }} \left( {0 {\text{T}}} \right)}} \times 100$ was measured at *f* = 500 kHz and *T* = 2 K along the *a*, *b*, and *c* axes (MD_*a*_ (Fig. 4a–c), MD_*b*_ (Fig. 4d–f), and MD_*c*_ (Fig. 4g–i) at *H*_*a*_, *H*_*b*_ and *H*_*c*_, respectively, up to ± 9 T. The MD curves vary in shapes and signs depending on the relative orientations of *ε'* and *H*. The full MD curves appear to be symmetric because the direction of each MD is indistinguishable in the applied AC electric field for the *ε'* measurements. MD_*a*_ varies slightly at *H*_*a*_ with the value of approximately 0.1% at 9 T (Fig. 4a), and it shows only a negligible *H*_*b*_ dependence (Fig. 4b). In Fig. 4c, a small peak in MD_*a*_ was observed at low *H*_*c*_, after which MD_*a*_ starts decreasing to a negative value with the change in slope at *H*_c_ ≈ 2.0 T and reaches approximately − 0.25% at 9 T. Applying both *H*_*a*_ and *H*_*b*_ (Fig. 4d,e), the initial curve of MD_*b*_ exhibits a small bending at low-*H* and tends to increase linearly by exhibiting a change in slope and maintaining a positive value throughout the range of *H*. MD_*b*_ at 9 T was found to be 0.32 and 0.74%, respectively, for *H*_*a*_ and *H*_b_. In contrast, the MD_*b*_ (Fig. 4f) tends to behave similarly to MD_*a*_ at *H*_*c*_ (Fig. 4c), with the maximum variation of − 0.65% at 9 T. Starting from the linear decrease upon increasing *H*_*a*_, MD_*c*_ changes in slope and shows a broad minimum at 3.7 T with − 0.4% variation (Fig. 4g). In Fig. 4h, MD_*c*_ begins to increase linearly at *H*_*b*_ ≈ 1.9 T and maintains the plateau mostly above 4 T. In Fig. 4i, the initial curve of MD_*c*_ increases with a slight curvature at low *H*_*c*_ regime and shows a kink at approximately 0.7 T, above which it reduces gradually, becomes almost linear above *H*_*c*_ = 2.2 T, and crosses zero at *H*_*c*_ ≈ 3.2 T. The maximum variation of MD_*c*_ is found to be approximately − 0.47% at 9 T.

**Figure 5:**
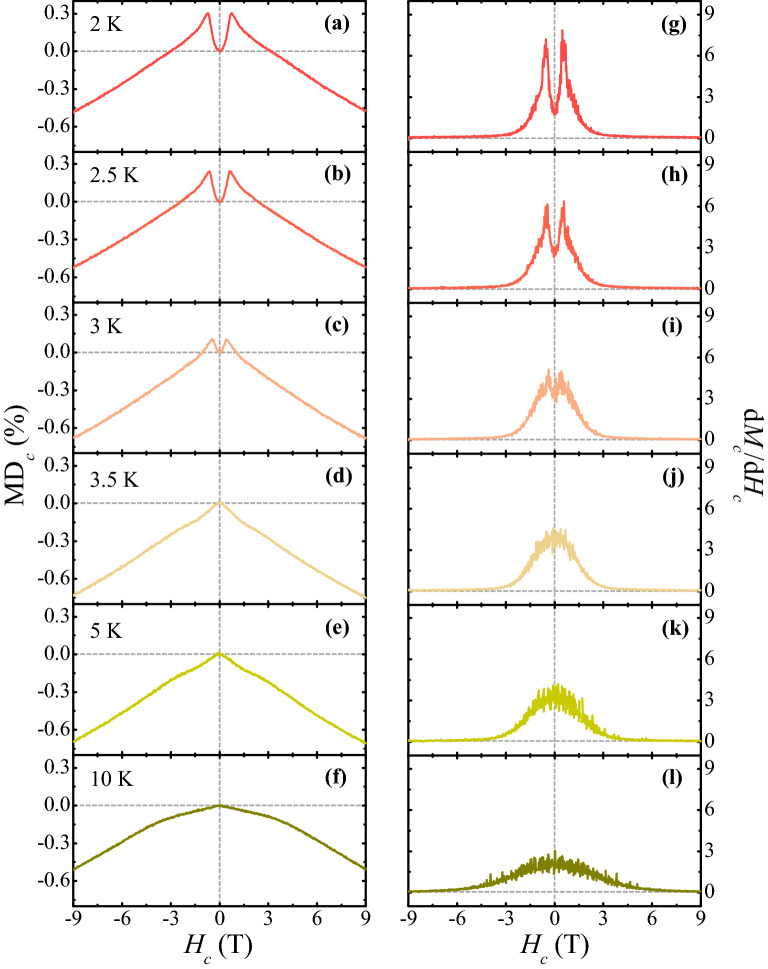
Temperature evolution of magnetodielectric effect along the *c* axis. (**a**)–(**f**) *H*_*c*_ dependence of MD_*c*_ at *T* = 2, 2.5, 3, 3.5, 5 and 10 K. (**g**)–(**l**) *H*_*c*_ derivative of *M*_*c*_ and d*M*_*c*_/d*H*_*c*_ measured at *T* = 2, 2.5, 3, 3.5, 5 and 10 K.

Among a variety of MD responses, as shown in Fig. 4, MD_*c*_ at *H*_*c*_ appears to be strongly correlated to the isothermal *M*_*c*_. The *T* evolution of MD_*c*_ examined this intriguing aspect at *H*_*c*_ compared with the *T* dependence of d*M*_*c*_/d*H*_*c*_. Figure 5 displays the *H*_*c*_-dependence of MD_*c*_ (Fig. 5a–f) and d*M*_*c*_/d*H*_*c*_ (Fig. 5g–l), measured up to ± 9 T at *T* = 2, 2.5, 3, 3.5, 5, and 10 K. Additionally, d*M*_*c*_/d*H*_*c*_ at 2 K is plotted in Fig. 5g for a precise comparison of the *H*_*c*_ dependence of MD_*c*_ at 2 K in Fig. 4a. d*M*_*c*_/d*H*_*c*_ demonstrates two sharp peaks, which coincide with the spin-flop transitions and sharp features in MD_*c*_. At 2.5 K, the characteristics of both MD_*c*_ and d*M*_*c*_/d*H*_*c*_ at 2 K are almost maintained (Fig. 5b,h). At 3 K, the anomalies in MD_*c*_, shown in Fig. 5c, are considerably diminished as small kinks along with the reduction of d*M*_*c*_/d*H*_*c*_ (Fig. 5i). In Fig. 5d, the kinks disappear at 3.5 K and a cusp occurs at *H*_*c*_ = 0 T. The value of MD_*c*_ at 9 T decreases continuously from − 0.47% at 2 K to − 0.74% at 3.5 K. At 5 and 10 K above *T*_Er_, the linear regime of MD_*c*_ is progressively curved with further suppression of d*M*_*c*_/d*H*_*c*_. The highly nonlinear *H*_*c*_-dependence of MD_*c*_ and its close correlation to d*M*_*c*_/d*H*_*c*_ below *T*_Er_ would be ascribed to a magnetostrictive effect. In EFO, the magnetostriction that results in the lattice contraction can occur due to the exchange couplings between the Er^3+^ and Fe^3+^ magnetic moments along the *c* axis below *T*_Er_. This may lead to a change in phonon energies, which are relevant to the displacement modes of Er^3+^ ions and consequently modifies *ε*_*c*_*'* based on the Lydanne-Sachs-Teller (LST) relation^32,33^.

Although a positive and negative MD has been previously presented^34^, it is unconventional that both the MD effects arise in a single-phase material such as EFO, depending on relative orientations between the electric and magnetic fields, as shown in Fig. 4. In addition to the possible magnetostrictive effect along the *c* axis, the spin-phonon couplings would be the possible cause for the versatile field dependences of the MD effects in EFO. As long-wavelength optical phonons are relevant for the frequency of *f* = 500 kHz used for the dielectric permittivity measurement, the spin-phonon coupling would be a plausible origin for the MD effects. The shift of optical phonon frequencies can be induced by the spin–spin correlation function as a result of the relation $\Delta {\upomega } \approx {\uplambda }\left\langle {S_{i} \cdot S_{j} } \right\rangle$^35,36^, where *λ* is the spin-phonon coupling constant and its typical value is known as a few cm^−1^ for the optical phonons in oxide materials^37–40^. Further, the spin–spin correlation function can be related to the magnetic contribution of heat capacity (*C*_m_) obtained after subtracting the phonon contribution proportional to *T*^3^:

$$\left\langle {S_{i} \cdot S_{j} } \right\rangle = \frac{1}{{8N_{A} J_{1} + 3N_{A} J_{2} }}\int {C_{m} \left( T \right)dT} ,$$

where 8*N*_A_ and *J*_1_ denote the number of bonds per mole and exchange constant for the Er-Fe pairs, respectively, and 3 *N*_A_ and *J*_2_ do the number of bonds per mole by considering the double counting and exchange constant for the Er-Er pairs, respectively^37^. Recent experiment of low-*T* Raman spectroscopy for the GdFeO_3_ reveals that Raman shift for the mode relevant to the motion of Gd^3+^ ions occurs below the *T*_Gd_ and is found to be ∆ω ≈ 1 cm^−1^^38^. In a theoretical work on the GdFeO_3_, *J*_1_ = 0.03 meV and *J*_2_ = 0.05 meV were also calculated^41^. Assuming similar results are expected in the EFO, the coupling constant based on the heat capacity data shown in Fig. 3b for the EFO was estimated as *λ* ≈ 3.2 cm^−1^. Moreover, close correlation between magnetic anisotropy and phonon spectra can be found in an example of Sr_4_Ru_3_O_10_^40^. Both increase and decrease of Raman shifts upon increasing magnetic fields were observed for the different field orientations. This implies that the spin correlations would be susceptible to the magnetic anisotropy and thus Raman shifts result in the positive or negative variations in a dielectric constant based on the LST relation. Our results motivate further optical experiments and theoretical studies to reveal the underlying mechanism for strongly anisotropic and nonlinear MD behaviors in EFO.

## Conclusion

In summary, we have synthesized single crystals of orthoferrite ErFeO_3_ and explored their magnetic and magnetodielectric properties along different crystallographic orientations. We demonstrate highly nonlinear magnetodielectric responses with both positive and negative effects, which is rare as well as significant in a single-phase material, depending on the relative orientations between the electric and magnetic fields. Furthermore, the simultaneous anomalies of the dielectric constant and magnetic-field derivative of magnetization, corresponding to the spin-flop transition, were observed with the electric and magnetic fields along the *c* axis below *T*_Er_ = 3.4 K. The symmetric exchange strictions, which act as mechanisms for multiferroicity and magnetoelectricity in GdFeO_3_ and DyFeO_3_, respectively, would be responsible for the magnetodielectric effect. The results of the intricate magnetodielectric properties demonstrated by ErFeO_3_ will encourage fundamental and applied research on magnetodielectric materials.

## Methods

Single crystals of EFO were grown by the flux method utilizing PbO, PbF_2_, PbO_2_, and B_2_O_3_ fluxes in a high-temperature furnace. Er_2_O_3_ and Fe_2_O_3_ powders were prepared in a stoichiometric ratio and mixed with the flux compound. The mixture was heated to 1,290 °C in a platinum crucible for 16 h until it was completely dissolved. Then, it was cooled slowly to 850 °C at a rate of 2 °C/h, and further cooled to room temperature at a rate of 100 °C/h. Large EFO crystals with a cuboid shape and a length up to approximately 1 cm on one side were obtained. The crystallographic structure of the EFO crystals was confirmed using an X-ray diffractometer (D/Max 2500, Rigaku Corp.). The oxygen vacancy of the EFO crystals was measured utilizing a WDS (Wavelength Dispersive X-ray Spectrometer) in an EPMA (Electronic Probe Micro-Analyzer, JEOL JXA-8530F). The incident electron beam was applied with an acceleration voltage of 15 kV and a current of 20 nA. The composition ratio was determined by analyzing the characteristic x-rays of each element measured in the four WDS channels with different wavelength ranges. The *T* and *H* dependences of the DC magnetization were measured using a vibrating sample magnetometer at *T* = 2–150 K and *H* = –9–9 T with a Physical Properties Measurement System (PPMS, Quantum Design, Inc.). The *T* dependence of specific heat was measured with the standard relaxation method in the PPMS. The *T* and *H* dependences of the dielectric constant were observed at *f* = 500 kHz using an LCR meter (E4980, Agilent).

## Supplementary information

Supplementary information.
