# Supplementary material for: Anisotropic and nonlinear magnetodielectric effects in orthoferrite ErFeO3 single crystals
Source: Sci Rep. 2020 Jul 16;10:11825. doi: 10.1038/s41598-020-68800-x (PMC7367349; doi:10.1038/s41598-020-68800-x)
Supplement: Supplementary file 1 — Supplementary information. [file 41598_2020_68800_MOESM1_ESM.pdf]

Supplementary Information for **Anisotropic and nonlinear magnetodielectric effects in orthoferrite $ErFeO_3$ single crystals**

Dong Gun Oh,[1] Jong Hyuk Kim,[1] Hyun Jun Shin,[1] Young Jai Choi,[1,*] and Nara Lee[1,*]

[1]*Department of Physics, Yonsei University, Seoul 03722, Korea*

Correspondence and requests for materials should be addressed to Y. J. C. (phylove@yonsei.ac.kr) or N. L. (eland@yonsei.ac.kr).

S1. Characterization of crystallographic structure for $ErFeO_3$ (EFO)

The crystallographic structure and absence of a second phase were checked by the Rietveld refinement using the FullProf program for the power X-ray diffraction data. The data were obtained with a Rigaku D/Max 2500 powder X-ray diffractometer using Cu-K_α radiation. The result suggests that the EFO crystallize in an orthorhombic perovskite with the *Pbnm* space group. The lattice constants are found to be a = 5.2611 Å, b = 5.5835 Å, and c = 7.5915 Å with the reliability factors; χ^2 = 3.29, R_p = 6.95 %, R_{wp} = 6.39 %, and R_{exp} = 3.52 %. Further details of crystallographic data are summarized in Table S1.

Table S1. Unit cell parameters, reliability factors, and position parameters for EFO.

	**$ErFeO_3$**
**Structure**	Orthorhombic
**Space group**	*Pbnm*
**Lattice parameters/Å**	a = 5.2611(1)<br>b = 5.5835(1)<br>c = 7.5915(2)<br>c/a = 2.732
**R-factors/%**	R_p = 6.95<br>R_{wp} = 6.39<br>R_{exp} = 3.52
χ^2	3.29

**Er (x, y, z)**	(0.98127, 0.06922, 0.25)
**Fe (x, y, z)**	(0, 0.5, 0)
**O_1 (x, y, z)**	(0.1139, 0.4588, 0.25)
**O_2 (x, y, z)**	(0.695, 0.3085, 0.0597)

S2. Absence of ferroelectricity in EFO

The absence of ferroelectricity in EFO is evidenced by zero pyro- and magneto-electric currents. In Fig. S1 (a), the pyroelectric current was obtained by the temperature (*T*) variation of 4 K/min after poling in a static electric field of E_c = 4.9 kV/cm. Compared with the sharp anomaly at T_{Er} = 3.4 K for the *T* dependence of the magnetic susceptibility along the *c* axis, zero value of the pyroelectric current clearly indicates that the EFO crystal is not a ferroelectric. To confirm further, we also measured the magnetoelectric current with the magnetic field variation of 0.01 T/s at 2 K after poling in static electric and magnetic fields, E_c = 4.9 kV/cm and H_{c} = 9 T (Fig. S1(b)), which exhibits no sign of ferroelectricity or magnetoelectricity.

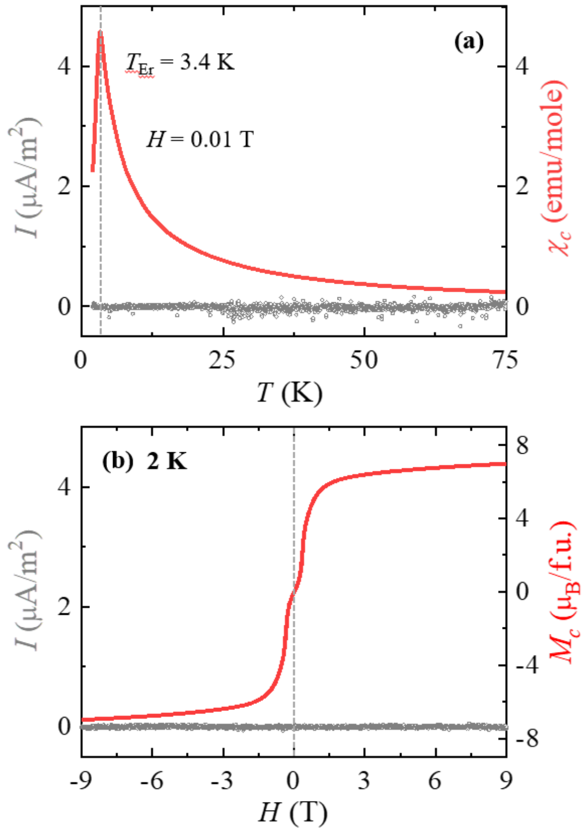

Figure S1. Absence of ferroelectricity in $ErFeO_3$. (a) Pyroelectric current measured upon warming after poling in E_c = 4.9 kV/cm. Temperature dependence of magnetic susceptibility for H = 0.01 T upon warming after zero-magnetic-field cooling along the c axis. (b) Magnetoelectric current along the c axis, measured up to ±9 T in $H//c$ at 2 K. Isothermal magnetization in $H//c$.
